# The effect of hormone replacement therapy on the survival of UK women: a retrospective cohort study 1984−2017

**DOI:** 10.1111/1471-0528.17008

**Published:** 2021-11-25

**Authors:** N Akter, E Kulinskaya, N Steel, I Bakbergenuly

**Affiliations:** ^1^ School of Computing Sciences University of East Anglia Norwich UK; ^2^ Norwich Medical School University of East Anglia Norwich UK

**Keywords:** Hormone replacement therapy, menopause, mortality, primary care records, The Health Improvement Network

## Abstract

**Objective:**

To estimate the effect of estrogen‐only and combined hormone replacement therapy (HRT) on the hazards of overall and age‐specific all‐cause mortality in healthy women aged 46–65 at first prescription.

**Design:**

Matched cohort study.

**Setting:**

Electronic primary care records from The Health Improvement Network (THIN) database, UK (1984−2017).

**Population:**

105 199 HRT users (cases) and 224 643 non‐users (controls) matched on age and general practice.

**Methods:**

Weibull‐Double‐Cox regression models adjusted for age at first treatment, birth cohort, type 2 diabetes, hypertension and hypertension treatment, coronary heart disease, oophorectomy, hysterectomy, body mass index, smoking and deprivation status.

**Main outcome measures:**

All‐cause mortality.

**Results:**

A total of 21 751 women died over an average of 13.5 years follow‐up per participant, of whom 6329 were users and 15 422 non‐users. The adjusted hazard ratio (HR) of overall all‐cause mortality in combined HRT users was 0.91 (95% CI 0.88−0.94), and in estrogen‐only users was 0.99 (0.93−1.07), compared with non‐users. Age‐specific adjusted HRs for participants aged 46–50, 51–55, 56–60 and 61–65 years at first treatment were 0.98 (0.92−1.04), 0.87 (0.82−0.92), 0.88 (0.82−0.93) and 0.92 (0.85−0.98) for combined HRT users compared with non‐users, and 1.01 (0.84−1.21), 1.03 (0.89−1.18), 0.98 (0.86−1.12) and 0.93 (0.81−1.07) for estrogen‐only users, respectively.

**Conclusions:**

Combined HRT was associated with a 9% lower risk of all‐cause mortality and estrogen‐only formulation was not associated with any significant changes.

**Tweetable abstract:**

Estrogen‐only HRT is not associated with all‐cause mortality and combined HRT reduces the risks.

## Tweetable abstract

Estrogen‐only HRT is not associated with all‐cause mortality and combined HRT reduces the risks.

## Introduction

Hormone replacement therapy (HRT) is an effective treatment for perimenopausal symptoms.^1^ Other known benefits include reduced osteoporosis and cardiovascular disease, and improved quality of life after menopause.^2^, ^3^, ^4^ A major meta‐analysis^5^ published in 2019 reported an increased risk of breast cancer associated with all types of HRT, and reports from the Million Women Study and an older meta‐analysis showed an increased risk of gynaecological cancer.^6^, ^7^, ^8^ Since then, many symptomatic women have been understandably cautious about taking HRT.

Past studies mostly focused on morbidity,^4^, ^7^, ^8^, ^9^, ^10^ or cause‐specific mortality,^11^, ^12^, ^13^ whereas all‐cause mortality summarises the net effects of HRT and is arguably a more useful single measure of the major risks and benefits over time. Previous observational studies of HRT and all‐cause mortality and a meta‐analysis comprising 16 000 women of mean age 55 years from 19 randomised trials, and 212 171 women from eight prospective cohorts found a reduced overall risk of death in HRT users.^14^, ^15^, ^16^, ^17^, ^18^ Pooled results from the Women’s Health Initiative’s (WHI) two trials showed no association of HRT with all‐cause mortality.^19^ Other surveys and long‐term cohort studies have variously reported no association between HRT and overall mortality^20^, ^21^ and increased risks of all‐cause mortality,^22^ and the authors have called for further research.

Clinical variables are important confounders that influence mortality, and hence adjustment for these factors is required to obtain a more accurate estimate of effect‐size and direction. Inclusion of healthy users compared with non‐users in some studies may have introduced bias in favour of HRT users.^14^, ^17^ The impact of estrogen‐only, and combined estrogen and progesterone formulations on all‐cause mortality has been reported in two papers,^19^, ^23^ where one found no association and the other found a reduced risk in younger users of combined HRT. The WHI^19^ results may not be generalisable to all users, as each trial assessed only one dose, formulation and route of administration of HRT. Other limitations of previous studies include the lack of age‐specific information on the use of HRT and its long‐term impact on all‐cause mortality,^2^, ^14^, ^17^, ^21^ and little information about the handling of missing data^14^, ^17^ or the presence of time‐varying hazards.^13^, ^16^, ^17^

A matched cohort study where the controls have the same age and background as cases and have similar health characteristics, with adjustment for confounding variables and a longer follow‐up, offers the potential to overcome some of the limitations in previous studies. Electronic primary care databases in the UK retain a wide range of information including comorbidities, treatment history, and some socio‐demographic factors with long‐term follow‐up over many years. Mortality registration is regularly updated in primary care as general practitioners (GPs) are informed of the death of patients registered with them.^24^ While there has been extensive research on HRT, no published study to date has investigated all‐cause mortality associated with HRT using UK primary care data.

The main aims of this study were to estimate the effect of estrogen‐only and combined HRT on the hazards of all‐cause mortality in a large cohort of healthy women broadly representative of the British population, and to analyse age‐specific effects of HRT initiation on mortality.

## Methods

### Design and setting

A population‐based matched cohort study was designed to estimate the effect of HRT on the hazards of all‐cause mortality using The Health Improvement Network (THIN) database. This database holds health information on anonymised patients in UK primary care dating back to the 1960s. THIN is representative of the UK general population in terms of demographics, prevalence of major medical conditions and mortality rates when adjusted for demographics and deprivation.^25^ Currently, THIN database retains longitudinal records of 17 million patients from over 770 GP practices, of which 3.1 million are actively registered, covering 6.2% of the UK population.^26^, ^27^

### Selection of cases

The study entry criteria for cases were the record of first oral or transdermal HRT prescription between 46 and 65 years of age. Cases were either estrogen‐only or combined HRT users. The British National Formulary (BNF) drug codes were used to identify patients on HRT.^28^ Cases were classified as combined HRT users if they received estrogen and progesterone either in a a single prescription or in two separate prescriptions.

### Selection of controls

Controls were non‐users of HRT or any type of drugs containing estrogen or progesterone at baseline. Controls were matched with cases in a ratio of one to up to three by age and general practice. The study entry date for controls was the first HRT prescription date for their matched cases.

### Inclusions and exclusions

Participants were eligible for the study if, at the time of study entry, they had been registered as an active patient for at least 1 year and their health records had been accessed at least once within the 10 years prior to their study entry date. To avoid bias due to ‘immortal periods’, actively registered patients who were prescribed HRT after the acceptable mortality reporting (AMR) date (a starting date from which practice recorded mortality was close to age‐ and sex‐standardised national mortality rates) of the corresponding general practice, were selected for the study. Patients with a previous history of any kind of cancer, acute myocardial infarction, serious heart failure, stroke (except transient ischaemic attack), chronic kidney disease (stage 3–5), dementia, premature ovarian insufficiency, surgically induced menopause before 45, and premature menopause were excluded from the study at baseline.

The analyses included patients who were born between 1921 and 1960 and started HRT at the selected age from 1984 until the study end date of 1 January 2017, and their matched controls. Participants were followed up from the date of first HRT prescription until death, or transfer out, or the study end date, whichever came first. Patients who were transferred out during the study period were no longer followed up, and their observation time was censored at that time. The SQL server 2016 was used to extract data from THIN.

### Variable selection

The covariates were selected based on their importance identified from past research, and expert knowledge within the team.^29^ The participants’ baseline characteristics were extracted from the latest records before the study entry date and included age at HRT prescription, birth year, type 2 diabetes, osteoporosis, peripheral arterial or vascular disease (PAD/PVD), coronary heart disease (CHD), hypertension and its treatments, hypercholesterolaemia, oophorectomy or hysterectomy, smoking, body mass index (BMI) and deprivation status. Information about parity (the number of previous births) and age at menopause was not included as it was not reliably recorded in the health records. Medical conditions were identified using the corresponding Read codes which are available online at ClinicalCodes.org.^30^

### Coding of covariates

Socio‐economic status in THIN is coded by the patient postcode‐based Townsend Deprivation Index, which is constructed from four census variables: households without a car, overcrowded households, households not owner‐occupied and persons unemployed. It is scaled from 1 to 5, where the first quintile represents the least deprived and the fifth represents the most deprived group.^31^ In the final analysis, patients within quintiles 1 and 2 were re‐coded as low, 3 as medium, and 4 and 5 as high deprivation. BMI was categorised as healthy weight or overweight, and obese. To classify hypertension, measurements of systolic and diastolic blood pressure (SBP ≥140 mmHg and DBP ≥90 mmHg) were used in conjunction with Read codes, as previous research has shown that using only Read codes to select hypertensive patients in THIN underestimates the actual prevalence of hypertension in the UK.^32^ Depending on the use of anti‐hypertensive drugs at baseline, hypertensive patients were categorised as treated or untreated. Uterine and ovarian status was grouped as intact (no history of removal of uterus and ovaries), hysterectomy with oophorectomy (hysterectomy and at least one ovary removed) or oophorectomy only (one or both ovaries removed). A very small group of women with hysterectomy without oophorectomy was not included in the model. Birth year was grouped into four decade‐long cohorts.

### Statistical analyses

The hazards of all‐cause mortality associated with HRT were initially estimated with a Cox proportional hazards regression model. The outcome was time in years from study entry to death from any cause. The model included second‐order interaction effects of all variables with the main exposure variable of HRT, and interactions of all medical conditions with the lifestyle variables. Backward elimination was applied to select the variables at 5% significance level for the main exposures, and 1% significance level for the interactions. The contribution of the covariates in explaining the variation of the hazard in the Cox regression model was assessed by analysis of variance (ANOVA). Grambsch and Therneau’s test^33^ was performed to check for non‐proportionality of hazards at a 5% level of significance and was found to be significant. The underlying baseline hazards of this study population were found to follow the Weibull distribution. Consequently, a model,^34^ which we refer to as Weibull‐Double‐Cox model, was fitted to estimate the shape parameters of the variables with time‐variant hazards, and the scale effects. In principle, this model replaced the unspecified baseline hazard in the Cox model by the Weibull hazard function and incorporates an additional Cox regression term for shape. General practices were included in the model as a random effect or frailty to account for unobserved heterogeneity of patients between practices. Four separate survival models were also fitted to assess the impact of HRT by 5‐year age group at initiation on all‐cause mortality. The same sets of explanatory variables were adjusted for in the full‐case (all age combined) model and in age subgroup analyses.

There were missing values for smoking, BMI, deprivation and hypertension status (Table S1). Multilevel multiple imputation (MI) was used to deal with missing data. Ten imputed datasets were generated and analysed independently for the full‐case model as well as for each subgroup model. It is widely accepted that when missingness varies from 10 to 50%, MI can be used to deal with missing data, and 5–10 imputations are sufficient, as having more imputations does not affect the results.^35^, ^36^ The distributions of the variables with missing records in complete and imputed datasets were similar (Table S2). Rubin’s rules^37^ were applied to pool the estimated parameters. Complete case analyses were performed to validate the imputation models (Figure S1). The overall performance of the models was assessed by the concordance, and its values of 0.7 in full model, and 0.75−0.81 in the subgroup models indicate a good fit.^38^ Kaplan–Meier (KM) survival analysis techniques were used to analyse the time to diagnosis of some selected medical conditions at follow‐up. All analyses were performed in statistical software R version 3.6.1 using the packages ‘survival’, ‘MASS’, ‘rms’ and ‘jomo’.

## Results

### Participants’ characteristics and follow‐up

**Figure 1:**
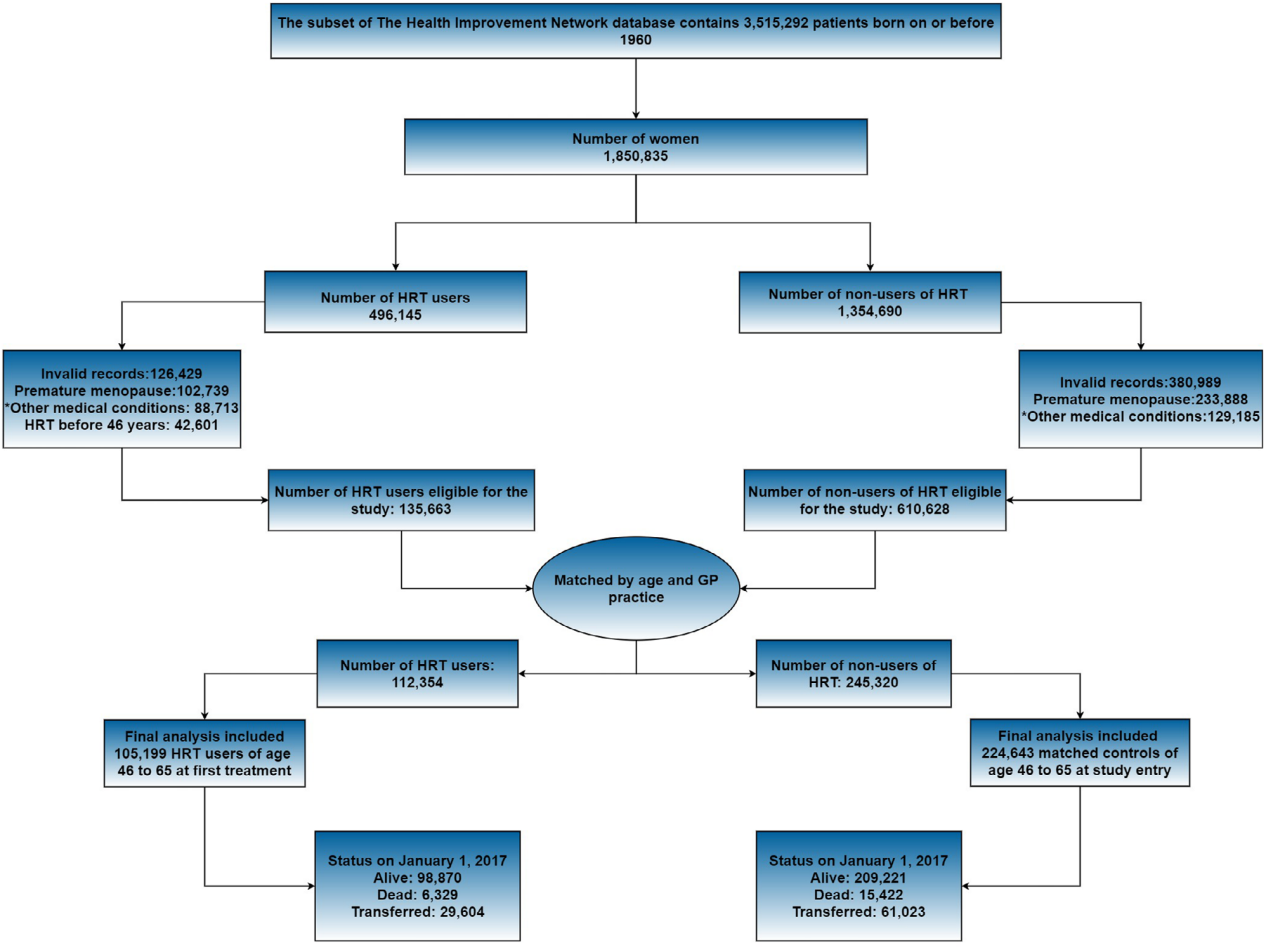
Selection procedure of study participants. Cases were HRT users and matched with controls by age and GP practice. Final analysis included patients who started treatment for the first time between 46 and 65 years of age, and their matched controls. *Other medical conditions that were excluded from the study were any kind of cancer, acute myocardial infarction, stroke, serious heart condition, chronic kidney disease and dementia.

Figure 1 shows the participant selection procedures, and the baseline characteristics of all study participants with follow‐up information are presented in Table 1. In all, 105 199 cases started treatment at age 46–65 years in 1984−2017, and there were 224 643 matched controls. The mean (± SD) age of all participants at first treatment was 53 (± 5.02) years. The mean duration of HRT use was 6.0 (± 4.8) years. Among cases, 17 606 (17%) received estrogen‐only and 87 593 (83%) received combined therapy. Around 75% of cases were prescribed first HRT between 46 and 55 years of age.

**Table 1:** Selected baseline characteristics and follow‐up information of cases and matched controls

Characteristic	No.(%) of patients**			
	Cases			Controls (*n* = 224 643)
	Estrogen‐only (*n* = 17 606)	Combined HRT (*n* = 87 593)	Total (*n* = 105 199)	
Death at follow‐up	1110 (6.3)	5219 (6.0)	6329 (6.0)	15 422 (7.0)
Transferred out	5078 (28.8)	24 526 (27.9)	29 604 (28.1)	61 023 (27.2)
Mean follow‐up years (± SD)	13.7 (7.1)	14.0 (6.7)	13.5 (6.8)	13.2 (7.0)
Age group at first HRT				
46–50	5035 (28.6)	37 219 (42.5)	42 254 (40.2)	87 108 (38.8)
51–55	6011 (34.1)	30 654 (35.0)	36 665 (34.9)	72 486 (32.3)
56–60	4069 (23.1)	13 286 (15.2)	17 355 (16.5)	40 674 (18.1)
61–65	2491 (14.1)	6434 (7.30)	8925 (8.5)	24 375 (10.9)
Birth cohort				
1921–1930	573 (3.3)	1361 (1.6)	1934 (1.8)	5565 (2.5)
1931–1940	5450 (31.0)	18 940 (21.6)	24 390 (23.2)	55 047 (24.5)
1941–1950	8438 (47.8)	44 453 (50.7)	52 891 (50.3)	96 142 (42.8)
1951–1960	3145 (17.9)	22 839 (26.1)	25 984 (24.7)	67 889 (30.2)
Hypertension				
No*	10 017 (56.9)	55 266 (63.1)	65 283 (62.1)	134 337 (59.8)
Treated*	4419 (25.1)	18 657 (21.3)	23 076 (22.0)	49 421 (22.0)
Untreated*	3170 (18.0)	13 670 (15.6)	16 840 (16.0)	40 885 (18.2)
Uterine/ovarian status				
Intact	6779 (38.5)	78 214 (89.3)	84 993 (80.8)	203 625 (90.6)
Hysterectomy with oophorectomy***	9945 (56.5)	1067 (1.2)	11 012 (10.5)	6502 (2.9)
Oophorectomy only	882 (5.0)	8312 (9.5)	9194 (8.7)	14 516 (6.5)
PAD/PVD	1348 (7.7)	7498 (8.6)	8846 (8.4)	17 340 (7.7)
Diabetes type 2	317 (1.8)	1233 (1.4)	1550 (1.5)	5089 (2.3)
CHD	336 (1.9)	1033 (1.2)	1369 (1.3)	3130 (1.4)
Osteoporosis	352 (2.0)	2101 (2.4)	2453 (2.3)	4215 (1.9)
Hypercholesterolaemia	254 (1.4)	972 (1.1)	1226 (1.2)	2605 (1.2)
Body mass index				
Healthy weight/overweight*	13 109 (74.5)	69 023 (78.8)	82 132 (78.1)	161 294 (71.8)
Obese*	4497 (25.5)	18 570 (21.2)	23 067 (21.9)	63 349 (28.2)
Smoking status				
Non*	10 966 (62.3)	50 716 (57.9)	61 682 (58.6)	141 301 (62.9)
Ex*	3187 (18.1)	15 854 (18.1)	19 041 (18.1)	35 269 (15.7)
Current*	3468 (19.7)	21 022 (24.0)	24 490 (23.3)	48 298 (21.5)
Deprivation status				
Low*	9648 (54.8)	47 738 (54.5)	57 386 (54.5)	117 488 (52.3)
Medium*	3662 (20.8)	17 957 (20.5)	21 616(20.5)	46 950 (20.9)
High*	4296 (24.4)	21 811 (24.9)	26 107 (24.8)	60 204 (26.8)

* The reported prevalence of variables with missing values are the mean of ten imputed datasets. Due to missingness in systolic and diastolic blood pressure, missing values were generated in hypertension category.

** All values are reported as *n* (%) except the mean follow‐up time.

*** Hysterectomy and at least one ovary removed.

Controls had more missing records than the cases. Incomplete medical records were more common in earlier birth cohorts, as expected from previous research that showed great improvement in recording after the initiation of the Quality and Outcomes Framework (QoF) in 2004.^39^ The prevalence of selected medical conditions was nearly the same for cases and controls at baseline. However, there were more oophorectomies and hysterectomies among cases (Table 1) and more participants with osteoporosis among first HRT starters in the older age group at baseline (Table S3). These conditions are more prevalent among HRT users, as these are often the cause of HRT treatment.^40^ There were more obese controls, compared with more healthy weight and overweight cases. The proportions of ex‐smokers and current smokers were slightly higher in cases. More than half of the participants had high socio‐economic status (Townsend index 1–2). In earlier birth cohorts, more women took estrogen‐only HRT, whereas in later birth cohorts, more women took combined HRT.

The length of study follow‐up was 32 years and the mean follow‐up of participants was 13.5 (SD ± 7.0) years. The interquartile range of participant follow‐up was 10.8 years. During follow‐up, 21 751 women died, of whom 6329 (6%) were cases and 15 422 (7%) were controls. In all, 44 cases died per 10 000 participant‐years follow‐up compared with 63 controls. During follow‐up, 29 604 (28%) cases and 61 023 (27%) controls were transferred out.

### Results of survival models

The covariates included in the final model were age at first HRT prescription, birth cohort, HRT type, hypertension and its treatment, CHD, type 2 diabetes, oophorectomy or hysterectomy, BMI, smoking and deprivation. All significant variables in the full model were also significant in all age subgroup models. There was no significant interaction of HRT with other variables, which means that the survival effect of HRT on the hazards of all‐cause mortality were the same across different subgroups.

**Figure 2:**
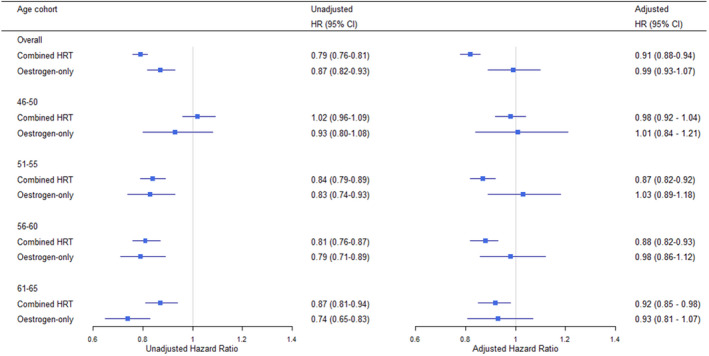
Unadjusted and adjusted hazard ratios of all‐cause mortality associated with the use of estrogen‐only and combined HRT. The age categories included patients who started HRT at that age and their matched controls. The hazard ratios (95% CI) were adjusted for age at first HRT, birth cohort, oophorectomy/hysterectomy status, type 2 diabetes, coronary heart disease, hypertension and its treatments, deprivation status, body mass index and smoking status. General practice was included in the model as frailty.

The adjusted hazard ratios for all‐cause mortality associated with HRT were time‐invariant. Overall, the hazard of death was lower in combined HRT users than in non‐users and there were no significant increased or decreased hazards associated with estrogen‐only HRT (Figure 2). The HR for combined HRT was 0.91 (95% CI 0.88–0.94) and for estrogen‐only users 0.99 (95% CI 0.93–1.07). In age subgroups, the HRs in combined HRT for women who received first treatment at age 46–50, 51–55, 56–60 and 61–65 were 0.98 (95% CI 0.92–1.04), 0.87 (95% CI 0.82–0.92), 0.88 (95% CI 0.82–0.93) and 0.92 (0.85–0.98), respectively. See Table S4 for all results.

Oophorectomy and hysterectomy were associated with improved survival prospects, in which the highest reduction of hazards was in the 61–65 age cohort and lowest in the 46–50 cohort (Figure S2). Both treated and untreated hypertension increased the hazards of all‐cause mortality and the findings did not differ substantially in the age subgroup models. Overall, living in more deprived areas was associated with 42% higher hazard of death than living in less deprived areas. The interaction of BMI and smoking also had a considerable impact on survival. The HRs of all‐cause mortality in current smokers compared with non‐smokers were higher in healthy weight and overweight women than in obese women in all age cohorts.

**Figure 3:**
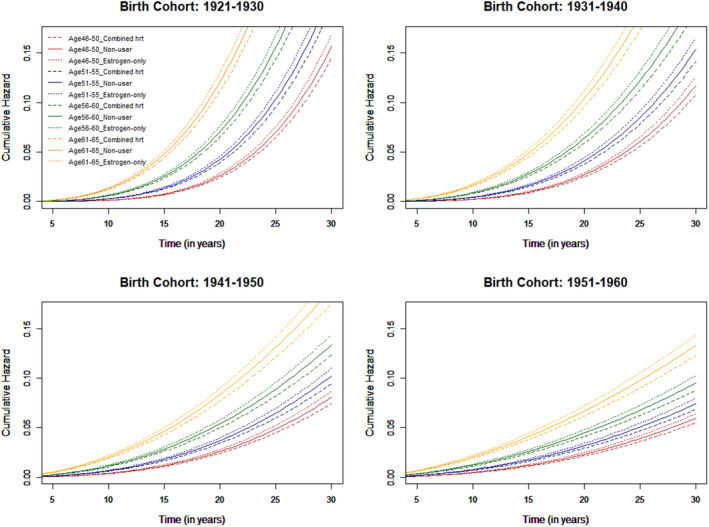
Cumulative hazards of all‐cause mortality associated with HRT for age subgroups of 46–50, 51–55, 56–60 and 61–65, respectively at first HRT treatment by HRT type in four birth cohorts.

As birth cohort was time‐variant, we calculated the cumulative hazards for each birth cohort, and found that longevity increased in women born in the later birth cohorts across all age and HRT type subgroups (Figure 3). Survival prospects also significantly varied by general practice with the variance of the frailty term 0.16 (95% CI 0.14–0.19) in the full model.

### Morbidity analysis at follow‐up

Hypertension and peripheral vascular disease were the commonest conditions to be diagnosed after study entry for both cases and controls (Table S5). The prevalence of hypertension was around 10% higher in estrogen‐only users than in non‐users. Osteoporosis was the next most common condition to be diagnosed in both groups. At follow‐up, cases had relatively more oophorectomies and hysterectomies. The prevalence of most chronic medical conditions was slightly higher in cases than in the controls, but there were more missing records in controls than in the cases.

The Kaplan–Meier plots (Figure S3) show that combined HRT users developed less type 2 diabetes at all time points over the entire follow‐up period. Until 10 years of follow‐up, there were no differences in heart failure prevalence among the groups. However, after 10 years, both estrogen‐only HRT users and non‐users developed more heart failure. Both combined HRT and estrogen‐only HRT users developed more breast cancer than the non‐users, but the proportion was slightly higher in the combined HRT group. There were lower probabilities of osteoporosis diagnosis among the combined HRT users after 10 years of follow‐up. There was no difference in the probabilities of dementia diagnosis among all groups up to 18 years of follow‐up. After 18 years, estrogen‐only HRT users developed slightly more dementia.

## Discussion

### Main findings

This large population‐based matched cohort study estimated the long‐term effects of HRT on the hazards of all‐cause mortality of 105 199 healthy women aged 46–65 years at first prescription compared with 224 643 matched healthy controls, using primary care data from 1984 to 2017. Our study found that during this long follow‐up, estrogen‐only HRT was not associated with significantly increased or decreased hazards of all‐cause mortality in any age group, and combined HRT was associated with a significantly decreased risk of death from all causes.

### Strengths and limitations

This study made use of electronic primary care records which are broadly representative of the UK general population.^25^ Availability of the information about prescribed medications in primary care records enabled us to select a large number of anonymised HRT users. The matched cohort study design and exclusion of the selected medical conditions from both cases and controls allowed us to estimate the effects of HRT on the survival of healthy users compared with healthy non‐users. The use of multiple imputation techniques for missing records allowed us to include nearly all extracted patients in the analyses. Use of the Weibull‐ Double‐Cox model enabled us to estimate the hazards of time‐variant covariates. A wide range of available information in primary care records including comorbidities, treatment history, lifestyle factors and demographics, allowed us to adjust for a high number of important confounders and the interaction between them. This study had an average patient follow‐up of almost 14 years.

The participants of this study received a wide variety of HRT preparations and doses, and therefore these were not differentiated in the analyses. Although many important risk factors were adjusted for, there is likely to remain residual confounding by a number of other risk factors, such as age at menopause, parity, diet and physical activity. These covariates were not adjusted for in the models as they were not reliably recorded in the health records. Duration of HRT use was not adjusted for, as it may potentially introduce immortality bias (longer use is confounded with longer survival). The higher rates of diagnosed conditions in HRT users compared with non‐users could be because the users visited the GP more frequently than the non‐users as they were receiving the treatment, and hence their health status was checked and updated more often. Although THIN is broadly representative of the UK general population, due to high geographical clustering in THIN,^41^ further research may be required to validate the results using data from other UK databases.

### Interpretation

The impact of estrogen‐only and combined HRT on all‐cause mortality was reported separately in only a few previous studies. The Women’s Health Initiative Trials^19^ found that combined or estrogen‐only HRT for a median of 5.6 years was not associated with all‐cause, cardiovascular or cancer mortality, and Stram et al.^23^ found a reduced risk of death in younger users of combined HRT but not in older postmenopausal women. Our results partly agree with these two studies but there were some major differences. WHI was a randomised control trial consisting of 13 816 postmenopausal HRT users versus placebo, and Stram et al.^23^ used survey data from the California Teachers Cohort Study. The mean age of women in both studies was around 63 years, which is more than a decade away from menopausal transition age. WHI investigated only one single dose of oral HRT, whereas the participants in this study took various doses and preparations of oral and transdermal HRT and were followed up for longer.

Pooled analysis of 26 708 women from 30 trials by Salpeter et al.^42^ showed that HRT reduced total mortality by 39% in women of mean age 54 years at baseline, but not in older women (mean age 66 years). Our results on combined HRT agree with Ettinger et al.^15^, Hunt et al.^16^, Grodstein et al.^17^ and Salpeter et al.,^18^ who also reported a reduced risk of all‐cause death in HRT users with a variation from 27 to 46%. However, this study found a lower reduction in hazards of death and several factors may have caused that difference. First, this study estimated hazards using health data from primary care, whereas most other studies used survey or register data. Secondly, we analysed combined and estrogen‐only HRT separately, while most other studies did not. Other possible causes of the lower reduction of hazards seen in this study in comparison with others is that the majority of observational studies did not have age‐matched controls, and some of them were criticised for healthy‐user selection bias.^17^ In this study, both cases and controls have the same age and similar health characteristics at study entry. In addition, this study estimated hazards of mortality by adjusting for a wide range of important confounders, whereas most other studies adjusted for demographic and/or lifestyle variables only. However, in unadjusted analysis, we found greater reduction of hazards of all‐cause mortality in both estrogen‐only and combined HRT users.

This study found no significant interactions of HRT type or age at initiation with other morbidities or lifestyle factors such as hypertension or smoking, which means that the effect of HRT on the hazards of all‐cause mortality were the same across different patient subgroups. This study found that a history of both oophorectomy and hysterectomy was associated with significantly improved survival. In addition, our results agree with the findings of Drever et al.^43^ with respect to significant survival variation due to deprivation. Finally, this study found significant heterogeneity in patients’ survival between general practices.

Current NICE clinical guidelines^44^ in the UK recommend offering combined HRT to symptomatic women with a uterus, and estrogen‐only HRT to women without a uterus after discussing the benefits and risks. According to the NICE, benefits of HRT include prevention of osteoporotic fractures, colorectal cancer, and cardiovascular disease if the therapy starts before the age of 60 years, and the risks include slight increase of CHD, stroke and thromboembolic events. All‐cause mortality studies have yet been not reviewed by NICE. In this study, combined HRT users had a lower incidence of type 2 diabetes, heart failure and somewhat less osteoporosis, and estrogen‐only users developed more hypertension and CHD events than the non‐users during follow‐up. Although the current NICE guideline states that estrogen‐only HRT is associated with little or no change in the risk of breast cancer and combined HRT can be associated with increased risk of breast cancer, we observed an increased incidence of breast cancer for both types of HRT. However, this did not translate into increased mortality in HRT users. It is therefore important for balanced information on the potential benefits and risks of HRT to be widely available to allow women and their GPs to make an informed choice.

## Conclusion

Compared with non‐users, we found combined HRT, but not estrogen‐only HRT, to be associated with a reduced risk of all‐cause mortality in a large population of healthy women followed up for many years. This information may assist women and their doctors in making decisions around HRT use. This research strengthens the emerging consensus that the benefits of long‐term HRT outweigh the harms for most women. However, each woman should make an informed decision about the likely risks and benefits, considering her own clinical condition, concerns and expectations.

### Disclosure of interests

None declared. Completed disclosure of interests form available to view online as supporting information.

### Contribution to authorship

NA performed statistical analyses, interpreted the results and drafted the manuscript. EK designed the study, provided guidance on the statistical methods and interpretation of the results and contributed to writing the manuscript. NS provided guidance on the methods and interpretation of results and contributed to writing the manuscript. IB extracted and prepared THIN data. All authors discussed the results, were involved in revisions, read and approved the final manuscript.

### Details of ethics approval

This study was approved by THIN Scientific Review Committee (16THIN095) on 27 September 2018.

### Consent for publication

The authors confirm this work is original and has not been published elsewhere.

### Funding

The work of NA, EK, NS, and IB was funded by The Institute and Faculty of Actuaries (N/A).

This research project was funded by the Actuarial Research Centre of the Institute of Faculty of Actuaries (IFoA). The funder had no role in study design, data extraction, analyses, interpretation of the results or decision to publish.

## Supporting information


**Figure S1.** Adjusted hazard ratios with 95% confidence intervals of all‐cause mortality associated with the covariates in both complete case and full data analysis for all age group combined.
**Figure S2.** Adjusted hazard ratios with 95% confidence intervals of oophorectomy and hysterectomy status, hypertension and its treatments, and deprivation status for full data and four age subgroups of 46‐50, 51–55, 56–60 and 61–65, respectively at first HRT prescription.
**Figure S3.** Kaplan–Meier survival estimates of estrogen‐only, combined HRT and non‐users by various medical conditions that were diagnosed at follow‐up.
**Table S1.** Number (%) of missing records in covariates by case and control status.
**Table S2.** Distribution of the covariates with missing values in the complete and imputed data.
**Table S3.** Baseline characteristics of the study population by age subgroups at first HRT prescription.
**Table S4.** Unadjusted and adjusted hazard ratios of all‐cause mortality associated with HRT, and the adjusted HRs for other covariates in complete case and full data analysis.
**Table S5.** Prevalence of selected medical conditions women developed at follow‐up.

Supplementary Material

Supplementary Material

Supplementary Material

Supplementary Material

## Data Availability

THIN data is made accessible via IQVIA under a sub‐licence or research agreement approved by THIN Scientific Review Committee. THIN is a registered trademark of Cegedim SA in the UK and other countries. Reference made to THIN database is intended to be descriptive of the data asset licensed by IQVIA.
