# Supplementary material for: The effect of hormone replacement therapy on the survival of UK women: a retrospective cohort study 1984−2017
Source: BJOG. 2021 Nov 25;129(6):994–1003. doi: 10.1111/1471-0528.17008 (PMC9298998; doi:10.1111/1471-0528.17008)
Supplement: Supplementary file 1 — Figure S1. Adjusted hazard ratios with 95% confidence intervals of all‐cause mortality associated with the covariates in both complete case and full data analysis for all age group combined. Figure S2. Adjusted hazard ratios with 95% confidence intervals of oophorectomy and hysterectomy status, hypertension and its treatments, and deprivation status for full data and four age subgroups of 46‐50, 51–55, 56–60 and 61–65, respectively at first HRT prescription. Figure S3. Kaplan–Meier survival estimates of estrogen‐only, combined HRT and non‐users by various medical conditions that were diagnosed at follow‐up. Table S1. Number (%) of missing records in covariates by case and control status. Table S2. Distribution of the covariates with missing values in the complete and imputed data. Table S3. Baseline characteristics of the study population by age subgroups at first HRT prescription. Table S4. Unadjusted and adjusted hazard ratios of all‐cause mortality associated with HRT, and the adjusted HRs for other covariates in complete case and full data analysis. Table S5. Prevalence of selected medical conditions women developed at follow‐up. [file BJO-129-994-s001.docx]

# **Table S1.** Number (%) of missing records in covariates by case and control status

**Cases Controls**

**Covariates Oestrogen-only Combined Total**

**(17606) (87593) (105199) (224643)**

BMI 3356(19.1) 14016(16.0) 17372(16.5) 62629(27.9)

Smoking 2111(12.0) 7422(8.5) 9533(9.1) 37909(16.9)

Townsend 1828(10.4) 9093(10.4) 10921(10.4) 26604(11.8)

Hypertension^*^ 4629(26.3) 18463(21.1) 23092(22.0) 71201(31.7)

^*^Missing values were generated in hypertension category due to missingness in SBP (29%) and DBP (31%) records.

**Table S2.** Distribution^*^ of the covariates with missing values in the complete and imputed data

| Covariates | **Complete data^**^**  **(108255)** | | |  | **Imputed data*****  **(329842)** | | |
| --- | --- | --- | --- | --- | --- | --- | --- |
|  |  |  |  |  |  |  |  |
|  | Oestrogen-only (6539) | Combined  (36296) | Controls  (65420) |  | Oestrogen-only (17606) | Combined  (87593) | Controls  (224643) |
|  |  |  |  |  |  |  |  |
| **Hypertension** |  |  |  |  |  |  |  |
| No | 3523(53.9) | 22552(62.1) | 37904(57.9) |  | 10021(56.9) | 55267(63.1) | 134276(59.8) |
| Treated | 1785(27.3) | 8031(22.1) | 15182(23.2) |  | 4416(25.1) | 18658(21.3) | 49329(22.0) |
| Untreated | 1231(18.8) | 5713(15.7) | 12334(18.9) |  | 3169(18.0) | 13668(15.6) | 41038(18.3) |
| **Deprivation** |  |  |  |  |  |  |  |
| Low | 3646(55.8) | 20001(55.1) | 35326(54.0) |  | 9648(54.8) | 52057(54.5) | 127761(52.3) |
| Medium | 1327(20.3) | 7388(20.4) | 13457(20.6) |  | 3662(20.8) | 19574(20.5) | 51088(20.9) |
| High | 1566(23.9) | 8907(24.5) | 16637(25.4) |  | 4078(24.4) | 23811(24.9) | 65596(26.8) |
| **Smoking** |  |  |  |  |  |  |  |
| Non | 4150(63.5) | 21058(58.0) | 41858(64.0) |  | 10968(62.3) | 55378(57.9) | 152996(62.9) |
| Ex | 1218(18.6) | 6892(19.0) | 10714(16.4) |  | 3186(18.1) | 17317(18.1) | 38079(15.7) |
| Current | 1171(17.9) | 8346(23.0) | 12848(19.6) |  | 3468(19.7) | 22914(24.0) | 52240(21.5) |
| **Body mass index** |  |  |  |  |  |  |  |
| Healthy weight/overweight | 4734(72.4) | 28371(78.2) | 46299(70.8) |  | 14170 (74.5) | 74740(78.8) | 172613 (71.8) |
| Obese | 1805(27.6) | 7925(21.8) | 19121(29.2) |  | 4840 (25.5) | 20067(21.2) | 67826(28.2) |

*Values are reported as number (%)

**Patients with the complete records only

***Full dataset with the imputed values for missing records. The reported values are the mean of ten imputed datasets.

**Table S3.** Baseline characteristics of the study population by age subgroups† at first HRT prescription.

| **Characteristics** | **46-50** | | |  | **51-55** | | |  | **56-60** | | |  | **61-65** | | |
| --- | --- | --- | --- | --- | --- | --- | --- | --- | --- | --- | --- | --- | --- | --- | --- |
|  | Oestrogen-only | Combined | Controls |  | Oestrogen-only | Combined | Controls |  | Oestrogen-only | Combined | Controls |  | Oestrogen-only | Combined | Controls |
| **Hypertension** |  |  |  |  |  |  |  |  |  |  |  |  |  |  |  |
| No | 3399(67.5) | 25831(69.4) | 60144(69.1) |  | 3531(58.7) | 19144(62.5) | 43364(59.8) |  | 2060(50.6) | 7257(54.6) | 20212(49.7) |  | 1079(43.3) | 2993(46.5) | 10530(43.2) |
| Treated | 932(18.5) | 6804(18.3) | 14539(16.7) |  | 1387(23.1) | 6539(21.3) | 15592(21.5) |  | 1208(29.7) | 3411(25.7) | 11052(27.2) |  | 855(34.3) | 1998(31.1) | 8055(33.1) |
| Untreated | 704(14.0) | 4584(12.3) | 12425(14.3) |  | 1093(18.2) | 4971(16.2) | 13530(18.7) |  | 801(19.7) | 2618(19.7) | 9410(23.1) |  | 557(22.4) | 1443(22.4) | 5790(23.8) |
| **Uterine/ovarian status** |  |  |  |  |  |  |  |  |  |  |  |  |  |  |  |
| Intact | 2231(44.3) | 34623(93.0) | 83158(95.5) |  | 2233(37.2) | 27112(88.5) | 65141(89.9) |  | 1412(34.7) | 11165(84.0) | 34928(85.9) |  | 903(36.3) | 5314(82.6) | 20398(83.7) |
| Hysterectomy with oophorectomy | 2579(51.2) | 286(0.8) | 870(1.0) |  | 3451(57.4) | 359(1.2) | 2119(3.0) |  | 2460(60.5) | 254(2.0) | 1974(4.9) |  | 1455(58.4) | 168(2.6) | 1539(6.3) |
| Oophorectomy only | 225(4.5) | 2310(6.2) | 3080(3.5) |  | 327(5.4) | 3183(10.4) | 5226(7.2) |  | 197(4.8) | 1867(14.1) | 3772(9.3) |  | 133(5.3) | 952(14.8) | 2438(10.0) |
| PAD/PVD | 317(6.3) | 2270(6.1) | 5139(5.9) |  | 390 (6.7) | 1992 (6.5) | 4494(6.2) |  | 317 (7.8) | 1009(7.6) | 2969(7.3) |  | 241(9.7) | 611(9.5) | 2242(9.2) |
| Diabetes Type 2 | 66(1.3) | 361(1.0) | 1158(1.3) |  | 98(1.6) | 451(1.5) | 1604(2.2) |  | 85(2.1) | 260(2.0) | 1300(3.2) |  | 68(2.7) | 161(2.5) | 1027(4.2) |
| CHD | 31(0.6) | 223(0.6) | 445(0.5) |  | 91(1.5) | 306(1.0) | 762(1.1) |  | 117(2.9) | 275(2.1) | 919(2.3) |  | 97(3.9) | 229(3.6) | 1004(4.1) |
| Osteoporosis | 65(1.3) | 409(1.1) | 784(0.9) |  | 80(1.5) | 429(1.4) | 942(1.3) |  | 98(2.9) | 79(3.2) | 358(2.7) |  | 107(4.3) | 302(4.7) | 658 (2.9) |
| Hypercholesterolaemia | 372(7.4) | 2619(7.0) | 4299(5.0) |  | 464(7.7) | 2258(7.4) | 4468(6.2) |  | 365(9.0) | 1060(8.0) | 3025(7.4) |  | 217(8.7) | 554(8.6) | 1771(7.3) |
| **Body mass index** |  |  |  |  |  |  |  |  |  |  |  |  |  |  |  |
| Healthy weight/overweight | 3714(73.7) | 29193(78.4) | 63101(72.4) |  | 4420(73.5) | 24045(78.4) | 51277(70.7) |  | 3045(74.8) | 10621(79.9) | 28979(71.3) |  | 1926(77.3) | 5160(80.0) | 17533(71.9) |
| Obese | 1321(26.2) | 8026(21.5) | 24007(27.5) |  | 1591(26.5) | 6609(21.5) | 21209(29.3) |  | 1024(25.2) | 2665(20.1) | 11695(28.7) |  | 565(22.7) | 1274(19.8) | 6842(28.1) |
| **Smoking status** |  |  |  |  |  |  |  |  |  |  |  |  |  |  |  |
| Non | 2953(58.6) | 20360(54.7) | 55149(63.3) |  | 3736(62.1) | 18185(59.3) | 45164(62.3) |  | 2621(64.4) | 8121(61.1) | 25405(62.5) |  | 1657(66.5) | 3986(61.9) | 15538(63.7) |
| Ex | 828(16.4) | 6179(16.6) | 12291(14.1) |  | 1032(17.2) | 5682(18.5) | 11488(15.8) |  | 788(19.4) | 2639(19.8) | 6862(16.9) |  | 510(20.5) | 1383(21.5) | 4507(18.5) |
| Current | 1254(24.9) | 10680(28.7) | 19668(22.6) |  | 1243(20.7) | 6787(22.1) | 15834(21.8) |  | 660(16.2) | 2526(19.0) | 8407(20.7) |  | 324(13.0) | 1065(16.5) | 4330(17.7) |
| **Deprivation status** |  |  |  |  |  |  |  |  |  |  |  |  |  |  |  |
| Low | 2766(55.0) | 20129(54.1) | 46550(53.4) |  | 3331(55.4) | 16901(55.1) | 37981(52.4) |  | 2202(54.1) | 7334(55.2) | 20809(51.2) |  | 1373(55.1) | 3490(54.2) | 12275(50.4) |
| Medium | 989(19.6) | 7461(20.1) | 17679(20.3) |  | 1257(20.9) | 6241(20.4) | 15055(20.8) |  | 839(20.6) | 2717(20.5) | 8462(20.8) |  | 506(20.3) | 1307(20.3) | 5198(21.3) |
| High | 1280(25.4) | 9629(25.8) | 22879(26.3) |  | 1423(23.7) | 7512(24.5) | 19450(26.8) |  | 1028(25.3) | 3235(24.3) | 11403(28.0) |  | 612(24.6) | 1637(25.4) | 6902(28.3) |

†Each subgroup included patients who started HRT within that age range and their matched controls

**Table S4.** Unadjusted and adjusted hazard ratios of all-cause mortality associated with HRT, and the adjusted HRs for other covariates in complete case and full data analysis†

**Complete Case Analysis** **Full Data Analysis**

**Age groups** **Unadjusted Adjusted Unadjusted Adjusted**

HR (95% CI) HR (95% CI) HR (95% CI) HR (95% CI)

**HRT treatment**

Combined HRT 0.82(0.78-0.87)^*^ 0.92(0.87-0.97)^*^ 0.79(0.76-0.81)^*^ 0.91(0.88-0.94)^*^

Oestrogen-only 0.98(0.88-1.08)^*^ 1.08(0.95-1.13)^*^ 0.87(0.82-0.93)^*^ 0.99(0.93-1.07)^*^

**Hypertension**

**and its treatments**

Untreated -- 1.40(1.31-1.49)^*^ -- 1.31(1.24-1.38)^*^

Treated -- 1.55(1.46-1.65)^*^ -- 1.51(1.43-1.58)^*^

**Oophorectomy/**

**Hysterectomy status**

H+O†† -- 0.77(0.68-0.87)^*^ -- 0.76(0.71-0.81)^*^

Oophorectomy-only -- 0.89(0.81-0.96)^*^ -- 0.86(0.82-0.91)^*^

**Deprivation status**

Medium -- 1.23(1.15-1.31)^*^ -- 1.17(1.13-1.21)^*^

High -- 1.46(1.37-1.55)^*^ -- 1.42(1.38-1.47)^*^

**CHD**

Yes -- 1.58(1.41-1.78)^*^ -- 1.52(1.41-1.64)^*^

**Interaction of**

**BMI&Smoking**

H/O††† & ex-smoker -- 1.43(1.31-1.56)^**^ -- 1.41(1.33-1.52)^**^

H/O††† & current smoker -- 2.13(1.99-2.27)^**^ -- 2.10(2.00-2.25)^**^

Obese & non-smoker -- 1.30(1.21-1.41)^**^ -- 1.28(1.22-1.39)^**^

Obese & ex-smoker -- 1.62(1.48-1.78)^*^ -- 1.61(1.51-1.73)^*^

Obese & current smoker -- 2.19(2.03-2.73)^*^ -- 2.17(2.04-2.71)^*^

†Analysis included patients who started HRT within 46 to 65 years and their matched controls in both complete case and full data

††Hysterectomy with Oophorectomy

†††Healthy weight or overweight

^*^P-value<0.01

^**^P-value<0.001

**Table S5.** Prevalence of selected medical conditions women developed at follow-up

| **Medical conditions** | **No (%) of patients*^a^*** | | | |  |
| --- | --- | --- | --- | --- | --- |
|  | **Cases** | | **Controls** | *P*-value*^b^* | |
|  | Oestrogen-only  (*n* = 17606) | Combined HRT  (*n* = 87593) | (*n* = 224643) |  |  |
| Hypertension | 4893 (48.8) | 21701 (39.3) | 50866 (37.8) | 2.2e-16 | |
| PVD/PAD*^c^* | 3912 (24.1) | 19041 (23.8) | 39931 (19.3) | 2.2e-16 | |
| Osteoporosis | 2605 (15.1) | 12102 (14.2) | 30396 (13.8) | 8.3e-06 | |
| CKD*^d^* | 1957 (11.1) | 7649 (8.7) | 19665 (8.8) | 2.2e-16 | |
| Diabetes Type 2 | 1454 (8.4) | 5739 (6.7) | 17030 (7.8) | 2.2e-16 | |
| Hypercholesterolaemia | 1418 (8.2) | 6491 (7.5) | 13563 (6.1) | 2.2e-16 | |
| CHD*^e^* | 1227 (7.1) | 4582 (5.3) | 10327 (4.7) | 2.2e-16 | |
| Breast cancer | 723 (4.1) | 4172 (4.7) | 6243 (2.8) | 2.2e-16 | |
| Oophorectomy | 890 (16.7) | 18146 (23.4) | 23812 (12.0) | 2.2e-16 | |
| Hysterectomy | 586 (9.3) | 6893 (8.0) | 8308 (3.8) | 2.2e-16 | |
| Dementia | 516 (2.9) | 2222 (2.5) | 5042 (2.3) | 1.8e-11 | |
| Heart failure | 433 (2.4) | 1714 (2.0) | 5100 (2.3) | 3.2e-08 | |
| Myocardial infraction | 390 (2.2) | 1803 (2.1) | 4427 (2.0) | 4.0e-02 | |
| TIA*^f^* | 459 (2.6) | 1876 (2.1) | 3860 (1.7) | 2.2e-16 | |

*^a^*All values are reported as No. (%), percentages were calculated by the number of conditions patients developed at follow-up over the number of patients who did not have that condition at baseline.

*^b^P*-values are obtained from a *χ*^2^- test.

*^c^*Peripheral vascular/arterial disease *^d^*chronic kidney disease *^e^* coronary heart disease

*^f^*Transient ischaemic attack


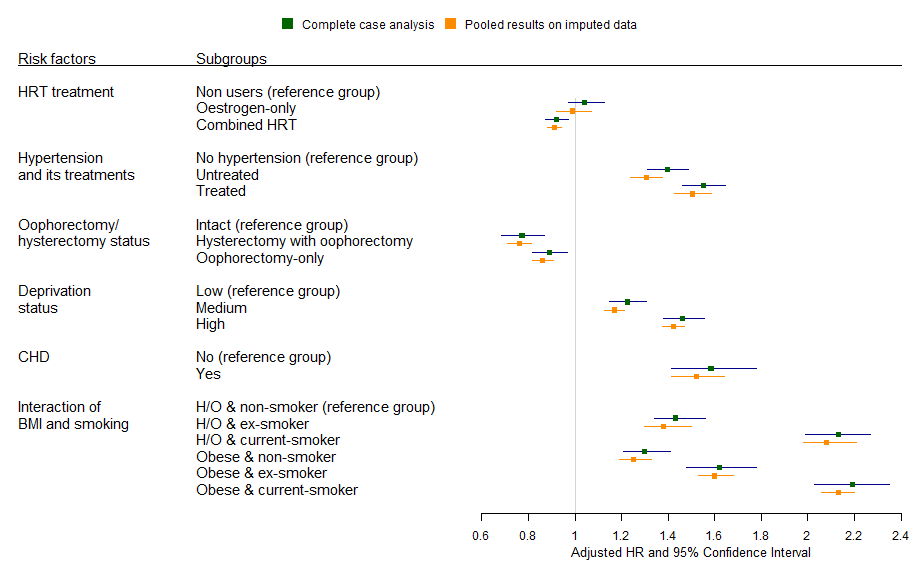


**Figure S1.** Adjusted hazard ratios with 95% confidence intervals of all-cause mortality associated with the covariates in both complete case and full data analysis for all age group combined.


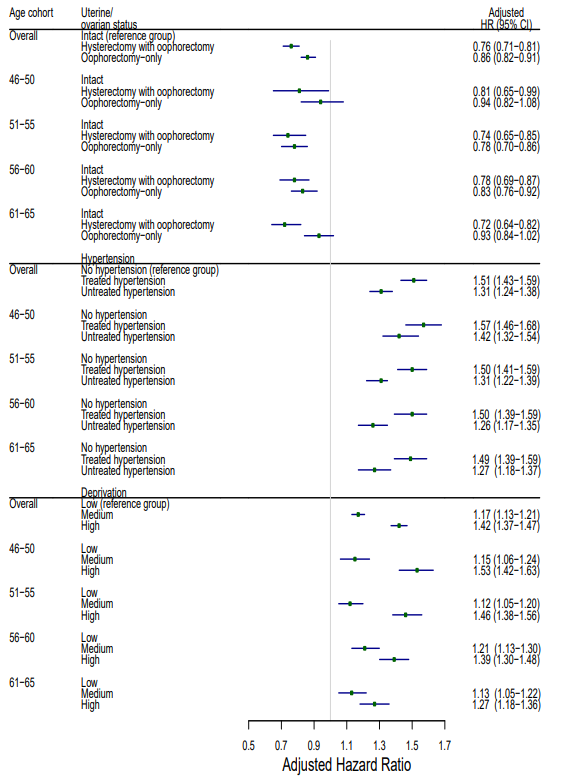


**Figure S2.** Adjusted hazards ratios and 95% confidence intervals of oophorectomy/ hysterectomy status, hypertension and its treatments, and deprivation status and all-cause mortality for all age combined, and four age subgroups of 46-50, 51-55, 56-60, and 61-65 respectively at first HRT prescription.


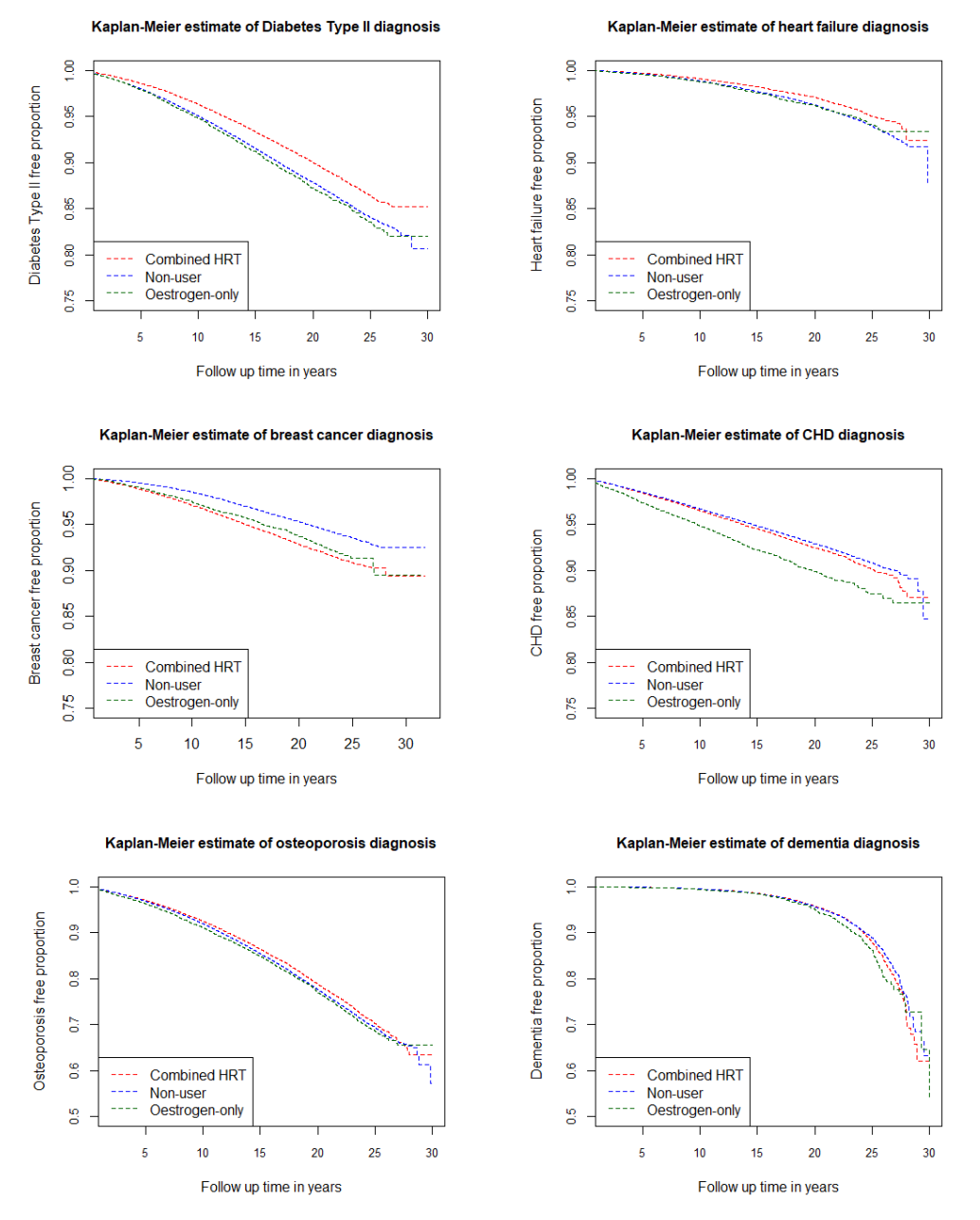


**Figure S3:** Kaplan-Meier survival estimates of oestrogen-only, combined HRT, and non-users by various medical conditions that were diagnosed at follow-up.
